# Development and Psychometric Evaluation of a Questionnaire to Assess Attitudes Towards Invisible Gender-Based Violence (Q-AIGV)

**DOI:** 10.1155/jonm/9977927

**Published:** 2024-12-09

**Authors:** Alba Fernández-Férez, Anabel Fernández-Vargas, Iria Dobarrio-Sanz, Amada Cesibel Ochoa-Pineda, Ximena Abarca-Duran, Ana Lucía Martínez-Abarca, Cayetano Fernández-Sola, José Manuel Hernández-Padilla

**Affiliations:** ^1^Critical Care Unit, Servicio Andaluz de Salud, Almería 04005, Spain; ^2^Department of Nursing Physiotherapy and Medicine, University of Almeria, Almeria 04120, Spain; ^3^Department of Geography, History and Humanities, University of Almeria, Almeria 04120, Spain; ^4^Faculty of Medical Sciences, University of Cuenca, Cuenca 010201, Ecuador; ^5^Researcher Pharmaceutical Public Health Unit, Institute of Tropical Medicine Antwerp, Antwerp, Belgium; ^6^Faculty of Health Sciences, Universidad Autónoma de Chile, Santiago 7500000, Chile

**Keywords:** gender-based microaggressions, invisible gender-based violence, questionnaire, validation study

## Abstract

**Objective:** To develop and psychometrically evaluate a questionnaire to assess attitudes towards invisible gender-based violence (Q-AIGV).

**Background:** Invisible gender-based violence is defined as discriminatory attitudes and beliefs towards women that are culturally accepted and normalised in society.

**Methodology:** This is a cross-sectional descriptive observational study. The development of the initial version of the questionnaire, a pilot study (*N* = 63) and a final validation study (*N* = 1264) were carried out. Reliability, validity, stability and readability were tested.

**Results:** Exploratory factor analysis revealed that the Q-AIGV is composed of 15 items distributed in three factors. Known-groups analysis detected significant differences in two groups with different characteristics (age and relationship duration). Criterion validity indicated the existence of a moderate and significant correlation (*r* = 0.469; *p* < 0.001) between the mean score of the Q-AIGV and the ‘Scale of Attitudes towards Gender-based Violence'. The Q-AIGV's content validity index (CVI-t = 0.96) and internal consistency (*α* = 0.906) were excellent.

**Conclusion:** The Q-AIGV showed very good results for reliability, validity, stability and readability. This suggests that the Q-AIGV could be a good tool for assessing attitudes towards invisible gender-based violence.

## 1. Introduction

Violence against women (VAW) is a gender-based violation of women's human rights [1]. It is a universal issue and a pressing public health problem, affecting the victim and her social circle [2]. VAW is a phenomenon that persists despite societies' attempts to eradicate it [3]. The efforts of certain societies to eliminate any violent or discriminatory attitudes [4] do not yield satisfactory results if we examine VAW figures [1]. In Spain, the percentage of women who were victims of gender-based violence in 2021 was 24% [1]. Around 30% of women have experienced gender-based violence worldwide [1].

The United Nations [5] defines VAW as ‘any act of gender-based violence that results in, or is likely to result in, physical, sexual, or mental harm or suffering to women, including threats of such acts, coercion or arbitrary deprivation of liberty, whether occurring in public or in private life'. A victim of VAW may suffer financial, psychological, physical, mental and sexual repercussions [6] and experience chronic conditions characterised by pain or fibromyalgia [7], negative attitudes towards self-health, such as smoking or drug abuse [8], gastrointestinal disturbances [9] and reproductive health disorders such as unwanted pregnancies or gynaecological disorders [10].

VAW stems from sexism [11], which is defined as the ideology that defends and justifies the superiority of men over women [12]. VAW is the most extreme manifestation of inequality between men and women in our society [13], but there are other types of subtle demonstrations that may go unnoticed, yet contribute to the manifestation of gender-based violence [14]. These are gender-based microaggressions or invisible gender-based violence (IGV), which can be defined as discriminatory attitudes and beliefs towards women that are culturally accepted, normalised and incorporated into society [15]. Although certain violent attitudes are more widely rejected, especially in Western societies [16], there are some gender-based microaggressions that often go unnoticed [17].

Bonino [18] developed a theoretical framework in which he defines IGV as ‘microaggressions' and ‘microviolence' that men use to maintain their position over women. They may not appear to be harmful and may even be normalised by society, but they are the breeding ground for gender-based violence [19]. The development of tools to identify attitudes towards IGV is therefore warranted. The development of valid and reliable tools to assess people's attitudes towards IGV could help healthcare professionals, social care workers and educators to assess the need for interventions aiming at reducing the risk of VAW behaviours in different populations. Therefore, the aim of this study was to develop and psychometrically evaluate a questionnaire to assess attitudes towards invisible gender-based violence (Q-AIGV).

## 2. Methodology

### 2.1. Design

An observational cross-sectional study was conducted in two phases. In the first phase, we developed the items comprising the Q-AIGV and conducted a pilot study to test its reliability (internal consistency) and content validity. In the second phase, we conducted a final validation study in which we assessed the validity, reliability and readability of the Q-AIGV.

### 2.2. Participants and Sample

This study was carried out at the University of Almeria between January 2021 and March 2022. Convenience sampling was used to select the sample. The inclusion criteria were (1) to be able to read and understand Spanish and (2) to be 18 years of age or over. A sample of 63 participants who did not participate in the final validation study was recruited for the pilot study. A sample of 1264 participants was recruited for the final validation study.

### 2.3. Ethical Considerations

The present study meets the Declaration of Helsinki's [20] criteria for biomedical research involving human beings. The project was approved by the Ethics and Research Commission of the Department of Nursing, Physiotherapy and Medicine at the University of Almería (EFM 141/2021). All participants were informed about the aim and the background of the study, the nature of their participation and how their data were going to be handled. The information section was presented at the beginning of the survey and included an agreement statement that those voluntarily willing to participate had to tick before accessing the different sections of the questionnaire. Once the survey was completed, the participants were presented with the following reminder by sending this response: “you are agreeing for your data to be used for research purposes only. Should you wish to withdraw from the study at any point after sending your response, please contact the principal researcher (JMHP)”. All participants' confidentiality and anonymity were guaranteed in accordance with European data protection law.

### 2.4. Data Collection

An online survey using the Google Forms tool was distributed through different social networks. The form that was disseminated had four sections. The first section included information about the study and the participants' rights. The second section was designed to collect sociodemographic data. The third section included the questionnaire that had been developed and needed to be tested. The fourth section presented the ‘Scale of Attitudes towards Gender-based Violence' [21], which is a 20-item measure of individual attitudes condoning different forms of gender-based violence. Although this tool measures more explicit forms of VAW, we selected it to test the Q-AIGV's criterion validity because some studies suggest that there is a relationship between invisible behaviours and the acceptability of more explicit sexist attitudes [22].

#### 2.4.1. Phase 1: Development and Pilot Study of the Q-AIGV

##### 2.4.1.1. Item Generation

In order to develop the items, a literature review was conducted first. The researchers decided to follow Bonino's theory of gender-based microaggressions [18] and Glick & Fiske's [19] theory of ambivalent sexism as a guide to develop the first version of the Q-AIGV. The initial version of the Q-AIGV was composed of 34 items with five response options, where 0 = *Strongly Disagree*, 1 = *Disagree*, 2 = *Neither Agree Nor Disagree*, 3 = *Agree* and 4 = *Strongly Agree*.

###### 2.4.1.1.1. Content Validity

A panel of 16 experts was formed who met the following inclusion criteria: (1) to have a degree in Health Sciences (Nursing, Psychology, Medicine), (2) to have more than 10 years of experience working in gender-based violence, (3) to have participated in the validation of previous questionnaires and (4) to have the literacy skills to read and write in Spanish. The 16 experts assessed the relevance of each of the items for measuring attitudes towards IGV. The method recommended by Polit & Beck [23] was followed. The experts had to rate each item as 1 = *Irrelevant*, 2 = *Somewhat Relevant*, 3 = *Relevant* and 4 = *Very Relevant* to assess attitudes towards IGV. The content validity index of each item (CVI-i) was calculated by adding the number of experts who rated an item as relevant or very relevant and dividing it by the total number of experts. Items with an I-CVI of 0.78 were considered acceptable [23].

##### 2.4.1.2. Pilot Study of the Q-AIGV

For the pilot study, the content validity and reliability of the Q-AIGV was analysed. In this phase of the study, the Q-AIGV was administered to a sample of 63 people and 16 experts not involved in the study.

###### 2.4.1.2.1. Reliability

To study the internal consistency of the Q-AIGV, three statistical parameters were analysed: (1) Cronbach's alpha of the scale (*α*), (2) corrected correlation of each item with the overall scale and (3) Cronbach's alpha of the scale when eliminating each item. Following the recommendations of international psychometric experts [23, 24] items were considered to contribute to good internal consistency of the Q-AIGV if they met the following criteria: (1) corrected item–total correlation (C-ITC) equal to or greater than 0.3 and (2) the total Cronbach's alpha of the scale did not increase significantly when removing the item. The Q-AIGV was interpreted as having good internal consistency if the total Cronbach's alpha of the scale was greater than 0.7 [21].

###### 2.4.1.2.2. Results of the Pilot Study

After this analysis, Items 6, 7, 8 and 24 were potentially subject to removal as they had a C-ITC of less than 0.3 and a significantly increased Cronbach's alpha when removed. However, the researchers decided to retain them for the final validation phase as they were theoretically consistent items, they were considered relevant by the experts and a pilot study was conducted with a relatively small sample size to make this decision (see Table 1).

#### 2.4.2. Phase 2: Final Validation Study

For the final psychometric analysis, the recommendations of experts in this field were followed [23, 24]. Statistical analysis was conducted using IBM SPSS Statistics 27 software. The validity and reliability of the Q-AIGV were assessed. In addition, readability was studied and a scoring system was developed to facilitate the interpretation of the results. A total of 1264 people participated in this phase of the study.

##### 2.4.2.1. Validity Analysis of the Final Version of the Q-AIGV

For the final validation of the Q-AIGV, construct validity, content validity and criterion validity were assessed.

###### 2.4.2.1.1. Construct Validity

Structural validity: An exploratory factor analysis (EFA) was carried out with a principal axis factoring and Varimax rotation. Prior to this, adequacy tests were carried out to evaluate the appropriateness of the analysis. An EFA was considered relevant when Bartlett's test of sphericity was significant (< 0.05) and the Kaiser–Meyer–Olkin index (KMO) was greater than 0.7 [23]. Likewise, three criteria were required to consider a factor as a dimension of the scale: (1) an eigenvalue greater than or equal to 1, (2) a factor loading of the items equal to or greater than 0.4 and (3) a clear graphical representation in the eigenvalue plot [21]. Items with factor loadings below 0.4 on all factors were removed from the Q-AIGV.

Known-groups analysis: The construct validity analysis was completed with known-groups analysis, for which participants' scores were compared according to age and length of time in a relationship with their intimate partner. For this, the Kruskal–Wallis test for K independent samples was used.

###### 2.4.2.1.2. Content Validity

The same steps were followed as for the pilot study. After calculating the CVI-i, the total CVI of the scale (CVI-t) was calculated. The CVI-i and the CVI-t were considered acceptable if greater than or equal to 0.78 [23].

###### 2.4.2.1.3. Convergent Validity

To assess its criterion validity, the participants' scores on the Q-AIGV were correlated to their scores on the ‘Scale of Attitudes towards Gender-based Violence' [21]. As a first step, normality tests were performed for the variable ‘Q-AIGV total score', with the following results: statistic = 0.50 (*df* = 1263), skewness (statistic = 0.65; SE = 0.69) and kurtosis (statistic = 0.377; SE = 0.138). Given that the variable ‘Q-AIGV total score' did not follow a normal distribution, Spearman's correlation coefficient (*r*) was calculated between the participants' scores in both questionnaires.

##### 2.4.2.2. Reliability of the Final Validation Study

The same steps were followed as in the pilot study. In addition, the stability of the scale was assessed by administering the Q-AIGV to a subsample (*n* = 53) twice with a 6-week interval between measurements. We analysed the test–retest reliability of the subscales that comprised the Q-AIGV by calculating the intraclass correlation coefficient (ICC).

##### 2.4.2.3. Readability

The INFLESZ scale was used to test the Q-AIGV's readability. This tool analyses the difficulty of the text and assigns a score from 0 to 100 points: < 40 points = *Very Difficult*, 40–55 points = *Somewhat Difficult*, 55–65 points = *Normal*, 65–80 points = *Quite Easy* and > 80 points = *Very Easy* [25]. Some sections were included where participants could provide suggestions, queries or difficulties encountered when reading the Q-AIGV.

##### 2.4.2.4. Scoring System

For the development of the internal scoring system [26], the maximum, minimum, mean and standard deviation of the total scores were taken into account. The range was calculated using the standard deviation score, and the starting point is set by the mean score. Subsequently, a standard deviation is subtracted or added to each category from the mean.

## 3. Results

### 3.1. Sample Description

In the final validation study, 1264 people participated, of whom 57.7% were women and 42.2% were men. The average age of the participants was 36.9 years (SD = 15.0). 83.2% of the participants stated that they were heterosexual, 5.5% bisexual and 3.4% homosexual. 67.6% had a partner. 45.7% said they were feminists, and 27.7% did not. 48% stated that they were Christians, and 43.6% were nonreligious. The rest of the sociodemographic data can be found in Table 2.

### 3.2. Validity

#### 3.2.1. Construct Validity

##### 3.2.1.1. EFA

According to the results of the Bartlett's test of sphericity (*x*2 (15223.660) = 561561; *p* < 0.001) and the KMO test (KMO = 0.945), it was appropriate to conduct an EFA. Six rounds of EFA were conducted. In the first EFA with the 34 items, it was observed that Items 5, 8, 14, 16 and 18 did not load on any factor (factor loadings < 0.40). In the second round of EFA, with the remaining 29 items, Items 6, 11, 21, 24, 26 and 32 were removed, in accordance with the previous criteria. In the third EFA, which was applied to the remaining 23 items, Items 7 and 12 had a factor loading of less than 0.4 and were therefore also removed. In the fourth round, only Item 13 was removed, in line with the aforementioned criteria. In the fifth round, Items 17, 22, 23, 29 and 30 were removed as their factor loadings were below 0.4. In the sixth and final round, the resulting 15 items that make up the final scale were distributed in three dimensions that represent 51.11% of the total variance explained. The 15-item version of the Q-AIGV had the following dimensional structure: (1) coercive and utilitarian attitudes (Items 3, 4, 9, 10, 15, 19 and 20), (2) crisis attitudes (Items 25, 27, 28, 31 and 33) and (3) ambivalent attitudes (Items 1 and 2). The EFA results of the final version of the Q-AIGV can be seen in Table 3.

##### 3.2.1.2. Known-Groups Analysis

The Kruskal–Wallis test was used to compare the participants' scores according to age and length of time in a relationship with their intimate partner. The participants' scores were significantly different depending on their age (*X*^2^ (4) = 17.134, *p*=0.002). It was also found that those who have been with their partners for more than 20 years tend to have more tolerant attitudes towards IGV than the rest of the groups (*X*^2^ (4) = 25.510, *p* < 0.001).

#### 3.2.2. Content Validity

The 15 items that comprised the final version of the Q-AIGV had a CVI-i above 0.78 (range 0.87–1). The CVI-t was 0.96 and was therefore considered excellent.

#### 3.2.3. Convergent Validity

The criterion validity analysis indicated the existence of a moderate and significant correlation (*r* = 0.469; *p* < 0.001) between the participants' mean score on the Q-AIGV and the ‘Scale of Attitudes towards Gender-based Violence' [21].

### 3.3. Reliability of the Scale

The reliability analysis of the final version of the Q-AIGV is shown in Table 4. All items showed a C-ITC of > 0.52. Cronbach's alpha of the scale does not increase if any item is removed. The final version of the Q-AIGV shows excellent internal consistency (*α* = 0.906). The stability analysis (*n* = 53) showed that the average measures ICC was 0.856 with a 95% confidence interval of 0.788–0.908 (*F* (52,364) = 6.93, *p* < 0.001). This suggests that the Q-AIGV is temporally stable.

### 3.4. Readability

Readability analysis suggests that the Q-AIGV is fairly easy to read (INFLESZ scale score = 68.47). The participants did not report any comprehension problems, so no modifications had to be made.

### 3.5. Scoring System

The mean total Q-AIGV score was 10 ± 9. This resulted in five categories to interpret according to how sexist the participants' attitudes were as follows: 0–9 = *Not at All Sexist*, 10–19 = *Slightly Sexist*, 20–29 = *Moderately Sexist*, 30–39 = *Highly Sexist* and > 40 = *Extremely Sexist*.

## 4. Discussion

The aim of this study was to develop and psychometrically evaluate Q-AIGV. The theoretical framework that guided the development of the scale was Bonino's theory of gender-based microaggressions and Glick and Flick's [19] theory of ambivalent sexism. Bonino [18] distinguishes three types of gender-based microaggressions: (1) coercive gender-based microaggressions, (2) covert gender-based microaggressions and (3) crisis gender-based microaggressions.

The questionnaire was subjected to an evaluation process in relation to validity, reliability and readability. This was carried out after a pilot study where, despite having items that could be removed, they were considered sufficiently relevant and consistent with the theoretical framework [18] to keep the 34 items for the final validation study. After performing all the calculations, 19 items were removed (Items 5, 6, 7, 8, 11, 12, 13, 14, 16, 17, 18, 21, 22, 23, 24, 26, 29, 30 and 32), resulting in Q-AIGV with 15 items.

The internal consistency of the Q-AIGV was excellent, indicating good reliability of the questionnaire [21]. The content validity was good, indicating that the items contributed to measuring the extent to which people agree with gender-based microaggressions being acceptable [18] or ambivalent sexism is tolerated [19]. To assess criterion validity, the correlation of the Q-AIGV with the Spanish version of Mateos [21] ‘Scale of Attitudes towards Gender-based Violence' was measured. The correlation found was moderately significant. This may be because the Q-AIGV measures attitudes towards more subtle forms of violence [18, 19]. Although for many authors, gender-based microaggressions are used to achieve the same end as other more explicit forms of gender-based violence [27], society tends to be more tolerant towards gender-based microaggressions [28]. Nonetheless, they are just as serious as they are an attack on women's autonomy and hinder the possibility of equal opportunities and work environments [29].

EFA was used to explore the Q-AIGV's construct validity, which allowed us to group the items into three dimensions based on Bonino's model. By means of the KGA, we were able to check whether the questionnaire was capable of detecting significant differences between groups who should have different attitudes a priori. Differences were found between groups according to age (grouped in intervals) and the length of time in a relationship with their intimate partner. People over 50 and those who have been with their partners for more than 20 years tend to have more tolerant attitudes towards IGV than the other groups. Other studies [30] also found a relationship between age and acceptance of gender-based microaggressions, indicating that the older the person, the more they displayed attitudes reflecting microaggressions. Similarly, García-Campana et al. [31] interviewed a group of adolescents, who agreed on the rejection of gender-based microaggressions. This suggests that young people tend to have less sexist attitudes than adults. This is consistent with studies suggesting that we still live in a society in which sexist attitudes persist and which the authoritative role of men is accepted [32].

### 4.1. Limitations

This study has limitations that must be taken into account when interpreting the results. First, the sample was obtained through convenience sampling, which requires the participants to be over 18 years of age. If participants of different age ranges had been included, this would have allowed us to obtain more intergenerational information. Another limitation is that the questionnaire was developed and tested amongst Spanish-speaking participants. The results cannot be generalised, and the Q-AIGV should be cross-validated before using it in different geographical and cultural settings. Furthermore, the questionnaire was carried out online and on social networks. As it was not carried out in person, it is not possible to ensure that participation was not influenced.

## 5. Conclusions

The psychometric assessment of the Q-AIGV showed good results. We conclude that this tool is reliable and valid for measuring attitudes towards IGV in the adult population. The use of the Q-AIGV in epidemiological studies with large and stratified samples could allow us to draw generalisable conclusions about attitudes towards IGV amongst Spanish-speaking populations. This could in turn be used as a basis for designing social and educational intervention strategies against IGV.

## Figures and Tables

**Table 1:** Psychometric properties of the pilot version of the Q-AIGV (*N* = 63).

	Cronbach's *α* if item deleted	C-ITC1	CVI-i2
Item 1. A woman should not be offended by someone making a sexual joke (e.g. about the number of people she has had sex with, about rape, or about sexual preferences)	0.839	0.415	0.93
Item 2. A woman should not be offended by someone making a joke about gender stereotypes (e.g. about women's abilities to do certain jobs, about their ability to drive or their suitability to carry out household chores, etc.)	0.839	0.415	1
Item 3. A man has the right to know where his partner is at all times	0.837	0.505	1
Item 4. A man should have access to his partner's mobile phone	0.839	0.543	1
Item 5. A woman's body is a legitimate advertising tool	0.839	0.403	0.81
Item 6. I don't think it's appropriate that only women feature in advertisements for detergents and household appliances etc	0.854	−0.106	0.93
Item 7. I believe that women are just as qualified as men to do any job (e.g. mining, firefighting, military, etc.)	0.853	−0.198	0.93
Item 8. I do not think it is appropriate to favour women in order to achieve equity in management positions in large companies and institutions (positive discrimination)	0.853	−0.042	0.93
Item 9. For certain things, a man's opinion should matter more (e.g. when buying a house or a car)	0.841	0.404	0.93
Item 10. For certain things, a woman's opinion should be more important (for example: decorating the house or choosing the children's school)	0.836	0.543	1
Item 11. I think it is correct to use the masculine as a generic term to refer to men and women (e.g. ‘actors' when addressing ‘actors' and ‘actresses')	0.845	0.228	0.87
Item 12. I do not think it is appropriate to feminise terms that do not exist (or are uncommon in English) in the feminine (e.g. ‘manageress' instead of ‘manager' or ‘vicaress' instead of ‘vicar'	0.844	0.235	0.93
Item 13. I think it is correct to use a neutral term, even if it does not exist in the dictionary (for example: ‘acters' to mean ‘actors/actresses')	0.844	0.245	1
Item 14. A man's deep voice is used to impose an opinion	0.841	0.346	1
Item 15. A man can make certain family decisions without consulting his partner (e.g. buying a new car etc.)	0.837	0.509	0.93
Item 16. It makes sense for women to take childcare leave if they have a lower salary	0.839	0.411	1
Item 17. If he really wants to achieve something, the man can insist until he convinces his partner by wearing her out	0.836	0.514	1
Item 18. Domestic work has no financial value, but rather family or social value	0.838	0.459	0.93
Item 19. Women are more able to provide care than men	0.840	0.390	0.93
Item 20. It makes sense for women to be the ones to take care of their children or loved ones	0.839	0.472	0.93
Item 21. Men getting angry in order to achieve something is a valid strategy in a relationship	0.846	0.194	1
Item 22. It is normal for a man to have to overrule his partner at times (for example, if she is too permissive with the children)	0.838	0.454	1
Item 23. The man must make the best decisions for the family or the couple even without consultation	0.839	0.507	1
Item 24. A man should not adopt a protective attitude without consulting his partner	0.852	−0.005	0.87
Item 25. A man can be coy to get something from his partner	0.837	−0.527	0.87
Item 26. It is not right for a man to withhold certain information from his partner (e.g. a female colleague inviting him to lunch, etc.)	0.849	−0.063	0.81
Item 27. It is fine to regale your partner for a particular purpose (e.g. before you tell him/her that you are going on a 2 week trip with friends)	0.835	0.580	1
Item 28. It is a valid strategy for a man to compare himself to worse men to make his partner value him more	0.835	0.589	1
Item 29. For a man to pretend to be absent-minded (e.g. by saying ‘I didn't notice') is a valid justification for harmful behaviour	0.835	0.611	1
Item 30. As a man, showing ‘moral support' to your partner is enough (e.g. supporting her to study or get promoted at work, but not taking on more household chores)	0.834	0.589	1
Item 31. A man has to let his partner do what she wants, even if he knows she is making a mistake, and then reproach her for it	0.837	0.495	0.93
Item 32. It is appropriate for a man to give gifts or make promises to a woman in order to gain an advantageous position	0.850	0.119	1
Item 33. It is right for a man to give in during an argument with his partner on occasion in order to benefit later on	0.837	0.518	1
Item 34. In the event of illness, however minor, it is logical for a man to try to be pitied in order to be looked after	0.838	0.477	1

^1^Corrected item–total correlation.

^2^Content validity index for each item.

**Table 2:** Sociodemographic data of the sample used in the final validation study.

Characteristics	Final validation study sample (*N* = 1264)
	*M* ± DE

Age	36.90 ± 15.011

	*n* (%)

*Gender*	
Female	729 (57.7)
Male	534 (42.2)
Other	1 (0.1)

*Sexual orientation*	
Heterosexual	1052 (83.2)
Homosexual	43 (3.4)
Bisexual	70 (5.5)
Other	1 (0.1)
Prefer not to declare	98 (7.8)

*Level of education completed*	
No studies	34 (2.7)
Primary	53 (4.2)
Secondary	178 (14.1)
High school	220 (17.4)
Vocational qualification	321 (25.4)
Undergraduate degree	325 (25.7)
Master's degree	130 (10.3)
Prefer not to declare	3 (0.2)

*Feminist*	
Yes	578 (45.7)
No	350 (27.7)
Prefer not to declare	336 (26.6)

*Monthly income*	
Low (less than €1000)	419 (33.1)
Medium (€1000–3000)	778 (61.6)
High (more than €3000)	64 (5.1)
Prefer not to declare	3 (0.2)

*Religion*	
Christian	611 (48.3)
Not religious	551 (43.6)
Muslim	72 (5.7)
Other	30 (2.4)

*Do you have a partner?*	
Yes	919 (72.7)
No	345 (27.3)

*How long have you been with your partner?*	
Less than 3 months	84 (6.6)
Between 3 months and 1 year	79 (6.3)
Between 1 and 3 years	166 (13.1)
Between 3 and 10 years	236 (18.7)
Between 10 and 20 years	155 (12.3)
More than 20 years	199 (15.7)

**Table 3:** Psychometric properties of the final validation study for the Q-AIGV (*N* = 1264).

	Cronbach's *α* if item deleted	C-ITC1	CVI-i2
*Ambivalent attitudes*			
Item 1. A woman should not be offended by someone making a sexual joke (e.g. about the number of people she has had sex with, about rape, or about sexual preferences)	0.900	0.597	0.93
Item 2. A woman should not be offended by someone making a joke about gender stereotypes (e.g. about women's abilities to do certain jobs, about their ability to drive or their suitability to carry out household chores, etc.)	0.901	0.582	1

*Coercive and utilitarian attitudes*			
Item 3. A man has the right to know where his partner is at all times	0.901	0.547	1
Item 4. A man should have access to his partner's mobile phone	0.899	0.651	1
Item 5. For certain things, a man's opinion should matter more (e.g. when buying a house or a car)	0.898	0.684	0.93
Item 6. For certain things, a woman's opinion should be more important (for example: decorating the house or choosing the children's school)	0.899	0.619	1
Item 7. A man can make certain family decisions without consulting his partner (e.g. buying a new car)	0.899	0.618	0.93
Item 8. Women are more able to provide care than men	0.903	0.523	0.93
Item 9. It makes sense for women to be the ones to take care of their children or loved ones	0.897	0.690	0.93

*Crisis attitudes*			
Item 10. A man can be coy to get something from his partner	0.900	0.586	0.87
Item 11. It is fine to regale your partner for a particular purpose (e.g. before you tell him/her that you are going on a 2-week trip with friends)	0.903	0.522	1
Item 12. It is a valid strategy for a man to compare himself to worse men to make his partner value him more	0.898	0.636	1
Item 13. A man has to let his partner do what she wants, even if he knows she is making a mistake, and then reproach her for it	0.902	0.537	0.93
Item 14. It is right for a man to give in during an argument with his partner on occasion in order to benefit later on	0.895	0.725	1
Item 15. In the event of illness, however minor, it is logical for a man to try to be pitied in order to be looked after	0.902	0.541	1

^1^Corrected item–total correlation.

^2^Content validity index for each item.

**Table 4:** Summary of exploratory factor analysis results of the Q-AIGV (*N* = 1264).

Items by factor	Factor		
	1	2	3
*Ambivalent attitudes*			
Item 1	0.275	0.267	0.747
Item 2	0.252	0.267	0.754

*Coercive and utilitarian attitudes*			
Item 3	0.489	0.288	0.164
Item 4	0.616	0.331	0.165
Item 5	0.749	0.251	0.177
Item 6	0.690	0.216	0.155
Item 7	0.583	0.265	0.200
Item 8	0.528	0.196	0.190
Item 9	0.670	0.311	0.197

*Crisis attitudes*			
Item 10	0.205	0.625	0.239
Item 11	0.173	0.563	0.221
Item 12	0.242	0.604	0.169
Item 13	0.373	0.453	0.122
Item 14	0.353	0.727	0.199
Item 15	0.340	0.485	0.096

## Data Availability

The data that support the findings of this study are available on request from the corresponding author. The data are not publicly available due to privacy or ethical restrictions.
